# Credentialing and retention of visa trainees in post-graduate medical education programs in Canada

**DOI:** 10.1186/s12960-017-0211-6

**Published:** 2017-06-12

**Authors:** Maria Mathews, Rima Kandar, Steve Slade, Yanqing Yi, Sue Beardall, Ivy Bourgeault, Lynda Buske

**Affiliations:** 1 Division of Community Health and Humanities, Memorial University of Newfoundland, St. John’s, NL Canada; 2 Canadian Post-MD Education Registry, Association of Faculties of Medicine of Canada, Ottawa, ON Canada; 3 Royal College of Physicians and Surgeons of Canada, Ottawa, ON Canada; 4 Health Canada, Government of Canada, Ottawa, ON Canada; 5 Telfer School of Management, University of Ottawa, Ottawa, ON Canada

**Keywords:** International medical graduate, Visa trainee, Post-graduate medical education, Retention, Physician supply

## Abstract

**Background:**

Visa trainees are international medical graduates (IMG) who come to Canada to train in a post-graduate medical education (PGME) program under a student or employment visa and are expected to return to their country of origin after training. We examined the credentialing and retention of visa trainees who entered PGME programs between 2005 and 2011.

**Methods:**

Using the Canadian Post-MD Education Registry’s National IMG Database linked to Scott’s Medical Database, we examined four outcomes: (1) passing the Medical Council of Canada Qualifying Examination Part 2 (MCCQE2), (2) obtaining a specialty designation (CCFP, FRCPC/SC), and (3) working in Canada after training and (4) in 2015. The National IMG Database is the most comprehensive source of information on IMG in Canada; data were provided by physician training and credentialing organizations. Scott’s Medical Database provides data on physician locations in Canada.

**Results:**

There were 233 visa trainees in the study; 39.5% passed the MCCQE2, 45.9% obtained a specialty designation, 24.0% worked in Canada after their training, and 53.6% worked in Canada in 2015. Family medicine trainees (OR = 8.33; 95% CI = 1.69–33.33) and residents (OR = 3.45; 95% CI = 1.96–6.25) were more likely than other specialist and fellow trainees, respectively, to pass the MCCQE2. Residents (OR = 7.69; 95% CI = 4.35–14.29) were more likely to obtain a specialty credential than fellows. Visa trainees eligible for a full license were more likely than those not eligible for a full license to work in Canada following training (OR = 3.41; 95% CI = 1.80–6.43) and in 2015 (OR = 3.34; 95% CI = 1.78–6.27).

**Conclusions:**

Visa training programs represent another route for IMG to qualify for and enter the physician workforce in Canada. The growth in the number of visa trainees and the high retention of these physicians warrant further consideration of the oversight and coordination of visa trainee programs in provincial and in pan-Canadian physician workforce planning.

## Background

Visa trainees are international medical graduates (IMG) who come to Canada to train in a post-graduate medical education (PGME) program under a student or employment visa [1, 2]. Visa trainees are funded by sponsors (usually foreign governments) that cover the trainee’s stipend and provide a fee (e.g., Can$100 000/year) to the training site or program [3–5]. All IMG, including visa trainees, must meet minimum qualifications to enter a PGME program including verification of qualifications, proof of language proficiency, and passing the Medical Council of Canada (MCC) Evaluating Examination and MCC Qualifying Examination Part 1. Visa trainees apply directly to training sites for a locally determined number of residency and fellowship positions [3, 5].

The goals of the visa training program are to meet Canadian training program needs, provide services, and/or fulfill Canada’s obligation to support medical training in less-developed countries [1, 2]. Given the number of IMG who migrate to Canada from developing countries, visa training programs represent one way in which Canada can contribute to the medical workforce of these source countries. Visa trainees are expected to return to their country of origin after completing their training once their student visas expire; however, many remain in Canada [1, 2, 6–8] on other types of visas that legally entitle them to work in Canada (e.g., temporary foreign worker visa) or by gaining immigrant status (e.g., permanent resident). Studies estimate that between 10% [9] and 20% [8] of visa trainees remain in Canada.

The number of PGME visa trainees in Canada has nearly doubled from 932 in 1985 to 1762 in 2014 [1, 10]. Despite their growing numbers, little is known about their PGME outcomes, specifically, their performance on examinations required for full licensure (the MCC Qualifying Examination Part 2 (MCCQE2) and specialty examinations from the Royal College of Physicians and Surgeons of Canada (RCPSC) or the College of Family Physicians of Canada (CFPC)) and whether writing these examinations is related to retention in Canada.

The purpose of this study is to examine the educational and retention outcomes of visa trainees in PGME programs in Canada. We describe the proportion and predictors of PGME visa trainees who (1) pass the MCCQE2, (2) pass specialty examinations, (3) work in Canada within 2 years of completing their PGME training, and (4) work in Canada in 2015. This study addresses a gap in the literature on the credentialing of visa trainees and their contribution to the supply of physicians in Canada. It evaluates the outcomes of a well-intentioned program that may nonetheless have unintended negative consequences.

## Methods

We linked data from the National IMG Database and Scott’s Medical Database. The National IMG Database captures longitudinal data to track IMG as they qualify for licensure and enter practice [6]. The Database includes data from various agencies that are involved in the training, assessment, certification, and licensing of IMG. Scott’s Medical Database is a listing of physicians in Canada who are members of the Canadian Medical Association and permit release of their information [11, 12]; it is the most comprehensive database available to track physician locations. As part of its ongoing reporting [13], the Canadian Post-MD Education Registry (CAPER) had data from Scott’s Medical Database for 2005 to 2015, with the exception of 2010 and 2014. Scott’s Medical Database does not keep historical data, so it was not possible to retroactively find location data for these 2 years.

We examined four outcomes: passed the MCCQE2 (Y/N), obtained a specialty designation (Y/N), work in Canada in the 2 years following training (Y/N), and work in Canada in 2015 (Y/N). Examination data were reported to the National IMG Database by the MCC, CFPC, and RCPSC. The National IMG Database records the year in which an IMG passes (or is exempt from) the MCCQE2 and the year in which they were awarded a specialty designation. It does not include whether an IMG wrote or failed the exam. In all our analyses, we assumed that all visa trainees would attempt to obtain credentials needed for full licensure. Visa trainees who were listed in Scott’s Medical Database within the first 2 years following their PGME training were considered to be working in Canada. The National IMG Database identified the last year of PGME enrollment, which we used to establish the 2 years of interest. We also identified whether the visa trainee was listed in the 2015 Scott’s Medical Database.

Other variables considered in the analysis were country group of medical school, gender (M/F), age at the start of PGME (under 30 years/over 30 years), years between MD and the start of PGME (younger graduate/older graduate), specialty (family medicine/other specialty), training region, and first rank (resident [PG1-7]/fellow [PG9]). The country of the trainee’s medical school was coded as one of five groups: Western (United Kingdom, Ireland, Western Europe, Australia, and New Zealand), Asia, the Caribbean and South America, the Middle East and North Africa, and others (Central and South Africa and Eastern Europe). We considered cultural similarity and size to create the five country groups and consulted with knowledge users (individuals who may use our research findings to inform policy decisions) to assess the face validity of the groupings. For the “years between MD and start of PGME” variable, we calculated the difference between the year of graduation from medical school and the year entering PGME, which we then coded as young graduate (−5 to 5 years between graduating from medical school and entering PGME) and older graduate (6+ years between graduating from medical school and entering PGME). Training region was coded as “Ontario” and “others” based on frequencies. We included residency and fellowship positions in our analyses because fellowships, by providing access to recognized clinical experience, enable some IMG to qualify for the MCCQE2 and specialty examinations. Fellowship applicants must have the same pre-requisites as applicants to residency programs (i.e., graduated from recognized medical school and passed/exempted from MCCEE and MCCQE1) [5].

For the retention outcomes, we also examined the variable “eligible for full license” (Y/N) which refers to whether a visa trainee had passed both the MCCQE2 and obtained a specialty designation.

To be included in the analyses, visa trainees had to have first entered a specialty PGME program in 2005 or 2006 or first entered a family medicine PGME program between 2005 and 2009. These cutoffs allow trainees sufficient time to qualify for and write the examinations and ensure that the passed exam would be recorded in the Database. Visa trainees who passed the MCCQE2 before 2005 (before the start of the Database) would not have the event recorded and would be coded as not passing the examination. The first and last years of PGME training are captured by CAPER and included in the National IMG Database.

Using SPSS (version 23.0), we described the characteristics of the sample and used chi-square tests between each outcome and relevant predictors. We used multiple logistic regression to identify significant (*P* < 0.05) predictors for each outcome. We selected potential predictors for each regression model on the basis of the chi-square tests. Variables were examined for possible co-linearity a priori. Predictors were removed from the model if they were not significant (based on the Wald test) and did not improve the −2 log likelihood value [14]. The tables list the variables included in the final regression models.

## Results

There were 233 visa trainees who met the inclusion criteria. The majority of visa trainees had graduated from a Middle Eastern or North African country (56.2%), was male (76.0%), was an older graduate (60.1%), and had entered a specialist PGME program (94.0%) (Table 1). Of the 219 visa trainees in specialist programs, 64 (29.2%) were in surgical specialty programs and 155 (70.8%) were in medical specialty programs (142 clinical specialties and 13 laboratory specialties) [15]. Just over one quarter (27.9%) was eligible to hold a full license (that is, passed both the MCCQE2 and a specialty examination).

**Table 1:** Characteristics of visa trainees who entered a family medicine PGME program between 2005 and 2009 or a specialty PGME program in 2005 or 2006

Variable	*n* = 233 *n* (%)
Passed MCCQE2	
Yes	92 (39.5)
No	141 (60.5)
Got specialty designation	
Yes	107 (45.9)
No	126 (54.1)
In Canada after training	
Yes	56 (24.0)
No	177 (76.0)
2015 location in Canada	
Yes	125 (53.6)
No	108 (46.4)
Country group of medical school	
Western	37 (15.9)
Asia	30 (12.9)
The Caribbean and South America	21 (9.0)
The Middle East and North Africa	131 (56.2)
Others	14 (6.0)
Gender	
Female	56 (24.0)
Male	177 (76.0)
Age at the start of PGME	
Under 30	76 (32.6)
30+	157 (67.4)
Years between MD and PGME	
Recent graduate (5 or less)	93 (39.9)
Older graduate (6+ years)	140 (60.1)
Specialty type	
Family medicine	14 (6.0)
Other specialty	219 (94.0)
First rank	
Resident	112 (48.1)
Fellow	121 (51.9)
Full license eligible	
Yes	65 (27.9)
No	168 (72.1)

*MCCQE2* Medical Council of Canada Qualifying Examination Part 2, *MD* medical degree, *PGME* post-graduate medical education

Less than half (39.5%) of visa trainees passed the MCCQE2. Compared to those who did not pass the MCCQE2 exam, a larger proportion of visa trainees who passed the MCCQE2 was in a family medicine program and a resident (Table 2). After controlling for other significant predictors, family medicine trainees were 8.33 times more likely than other specialist trainees to pass the MCCQE2 (Table 3). Residents were 3.45 times more likely than fellows to pass the examination.

**Table 2:** Visa trainees who passed and did not pass the MCCQE2 and who obtained and did not obtain a specialty designation among a cohort of visa trainees who entered a specialty PGME program in 2005 or 2006 or a family medicine PGME program between 2005 and 2009

Variable	Passed MCCQE2			Obtained specialty designation		
	Yes *n* (%)	No *n* (%)	*P* value	Yes *n* (%)	No *n* (%)	*P* value
Country group of medical school			0.095			0.007
Western	11 (12.0)	26 (18.4)		15 (14.0)	22 (17.5)	
Asia	13 (14.1)	17 (12.1)		12 (11.2)	18 (14.3)	
The Caribbean and South America	5 (5.4)	16 (11.3)		3 (2.8)	18 (14.3)	
The Middle East and North Africa	60 (65.2)	71 (50.4)		72 (67.3)	59 (46.8)	
Other	3 (3.3)	11 (7.8)		5 (4.7)	9 (7.1)	
Gender			0.365			0.018
Female	25 (27.2)	31 (22.0)		18 (16.8)	38 (30.2)	
Male	67 (72.8)	110 (78.0)		89 (83.2)	88 (69.8)	
Age at the start of PGME			0.154			0.000
Under 30	35 (38.0)	41 (29.1)		51 (47.7)	25 (19.8)	
30+	57 (62.0)	100 (70.9)		56 (52.3)	101 (80.2)	
Years between MD and PGME			0.086			0.001
Recent graduate (5 or less)	43 (46.7)	50 (35.5)		55 (51.4)	38 (30.2)	
Older graduate (6+ years)	49 (53.3)	91 (64.5)		52 (48.6)	88 (69.8)	
Specialty type			0.000			0.385
Family medicine	12 (13.0)	2 (1.4)		8 (7.5)	6 (4.8)	
Other specialty	80 (87.0)	139 (98.6)		99 (92.5)	120 (95.2)	
PGME region			0.992			0.804
Ontario	49 (53.3)	75 (53.2)		56 (52.3)	68 (54.0)	
Other	43 (46.7)	66 (46.8)		51 (47.7)	58 (46.0)	
First rank			0.000			0.000
Residents	62 (67.4)	50 (35.5)		79 (73.8)	33 (26.2)	
Fellows	30 (32.6)	91 (64.5)		28 (26.2)	93 (73.8)	

*MCCQE2* Medical Council of Canada Qualifying Examination Part 2, *MD* medical degree, *PGME* post-graduate medical education

**Table 3:** Predictors of visa trainees who passed the MCCQE2, and who obtained a specialty designation, among a cohort of visa trainees who entered a specialty PGME program in 2005 or 2006 or a family medicine PGME program between 2005 and 2009

Variable	Passed MCCQE2		Obtained specialty designation	
	OR (95% CI)	*P* value	OR (95% CI)	*P* value
Specialty type		0.009		NS
Family medicine	8.33 (1.69–33.33)		NS	
Other specialty	1.00		NS	
First rank		0.000		0.000
Residents	3.45 (1.96–6.25)		7.69 (4.35–14.29)	
Fellows	1.00		1.00	

*MCCQE2* Medical Council of Canada Qualifying Examination Part 2, *MD* medical degree, *PGME* post-graduate medical education, *OR* odds ratio, *95% CI* 95% confidence interval, *NS* not significant predictor (not included in the model)

Almost half the visa trainees (45.9%) obtained a specialty designation. Compared to those who did not get a specialty designation, a larger proportion of visa trainees who obtained a designation was a graduate from medical school in a Middle East or North African country, male, less than 30 when they started in a PGME program, a more recent graduate, and a resident (Table 2). Residents were 7.69 times more likely than fellows to obtain a specialty designation (Table 3).

Roughly one quarter (24.0%) of the visa trainees were working in Canada in the 2 years following the completion of their training program. Compared to visa trainees who did not work in Canada, a larger proportion of visa trainees who worked in Canada was eligible for a full license (Table 4). Visa trainees who were eligible for a full license were 3.41 times more likely to work in Canada in the 2 years following their PGME training than visa trainees who were not eligible for a full license (Table 5).

**Table 4:** Characteristics of visa trainees who worked and did not work in Canada after PGME training and in 2015, among a cohort of visa trainees who entered a specialty PGME program in 2005 or 2006 or a family medicine PGME program between 2005 and 2009

Variable	In Canada after training			In Canada in 2015		
	Yes *n* (%)	No *n* (%)	*P* value	Yes *n* (%)	No *n* (%)	*P* value
Country group of medical school			0.230			0.035
Western	4 (7.1)	33 (18.6)		22 (17.6)	15 (13.9)	
Asia	9 (16.1)	21 (11.9)		21 (16.8)	9 (8.3)	
The Caribbean and South America	6 (10.7)	15 (8.5)		15 (12.0)	6 (5.6)	
The Middle East and North Africa	35 (62.5)	96 (54.2)		59 (47.2)	72 (66.7)	
Other	2 (3.6)	12 (6.8)		8 (6.4)	6 (5.6)	
Gender			0.378			0.748
Female	11 (19.6)	45 (25.4)		29 (23.2)	27 (25.0)	
Male	45 (80.4)	132 (74.6)		96 (76.8)	81 (75.0)	
Age at start of PGME			0.371			0.006
Under 30	21 (37.5)	55 (31.1)		31 (24.8)	45 (41.7)	
30+	35 (62.5)	122 (68.9)		94 (75.2)	63 (58.3)	
Years between MD and PGME			0.606			0.001
Recent graduate (5 or less)	24 (42.9)	69 (39.0)		38 (30.4)	55 (50.9)	
Older graduate (6+ years)	32 (57.1)	108 (61.0)		87 (69.6)	53 (49.1)	
Specialty type			0.291			0.054
Family medicine	5 (8.9)	9 (5.1)		11 (8.8)	3 (2.8)	
Other specialty	51 (91.1)	168 (94.9)		114 (91.2)	105 (97.2)	
Training region			0.805			0.360
Ontario	29 (51.8)	95 (53.7)		70 (56.0)	54 (50.0)	
Other	27 (48.2)	82 (46.3)		55 (44.0)	54 (50.0)	
First rank			0.062			0.583
Residents	33 (58.9)	79 (44.6)		58 (46.4)	54 (50.0)	
Fellows	23 (41.1)	98 (55.4)		67 (53.6)	54 (50.0)	
Full license eligible			0.000			0.000
Yes	27 (48.2)	38 (21.5)		48 (38.4)	17 (15.7)	
No	29 (51.8)	139 (78.5)		77 (61.6)	91 (84.3)	

*MCCQE2* Medical Council of Canada Qualifying Examination Part 2, *MD* medical degree, *PGME* post-graduate medical education

**Table 5:** Predictors of visa trainees who worked in Canada after completing the PGME training and in 2105, among a cohort of visa trainees who entered a specialty PGME program in 2005 or 2006 or a family medicine PGME program between 2005 and 2009

Variable	Worked in Canada after training		Worked in Canada in 2015	
	OR (95% CI)	*P* value	OR (95% CI)	*P* value
Full license eligible		0.000		0.000
Yes	3.41 (1.80–6.43)		3.34 (1.78–6.27)	
No	1.00		1.00	

*MD* medical degree, *PGME* post-graduate medical education, *OR* odds ratio, *95% CI* 95% confidence interval, *NS* not significant predictor (not included in the model)

Over half (53.6%) of the visa trainees were working in Canada in 2015. More than 70% (170) of these visa trainees had exited their PGME program by 2010 and, by 2015, had remained in the country up to 10 years after their training. Compared to visa trainees who were not in Canada in 2015, a larger proportion of visa trainees who were in Canada in 2015 was over 30 years of age when they started their PGME program, and older graduate, and eligible for a full license (Table 4). Of the visa trainees who worked in Canada in 2015, 93 (74.4%) were in urban communities (population greater than 100 000), 25 (20.0%) were in small urban communities (population between 10 000 and 99 999), and 7 (5.6%) were in rural communities (population less than 10 000). Visa trainees who were eligible for a full license were 3.34 times more likely to work in Canada in 2015 than those who were not eligible for a full license (Table 5).

## Discussion

Visa trainees comprise a sizeable proportion of the IMG in PGME programs in Canada. In the National IMG Database, they represented 16.1% of the IMG who first entered specialty PGME programs in 2005 or 2006 or a family PGME program between 2005 and 2009. Compared to the 1214 non-visa IMG in this cohort, a smaller proportion of visa trainees than non-visa IMG passed the MCCQE2 (39.5% versus 92.8%), obtained a specialty designation (45.9% versus 73.2%), and were eligible for a full license (27.9% versus 71.3%) [16, 17]. We are unable to determine whether not realizing a milestone stems from visa trainees failing exams or not writing them. Visa trainees who did not intend to remain in Canada may not have written these exams. Some visa trainees may have passed the examinations outside the period captured by the National IMG Database (for example, 18.4% of the visa trainees in the study exited their PGME program after 2011).

These examinations are not mandatory for PGME trainees in Canada (visa trainee or otherwise), but passing the examinations is needed to obtain a full license that permits physicians to practice independently, without clinical supervision, and in the community of their choice. Qualifying for a full license would also facilitate immigration to Canada or other countries that recognize Canadian licensure; physicians who immigrate to Canada without these credentials would otherwise face lengthy delays in obtaining full credentials and employment in medicine. Almost two thirds of the visa trainees who remained in Canada following their training (51.8%) and in 2015 (61.6%) were not eligible for a full license and would likely have worked under a provisional (or restricted) license. While provisional licenses are used to recruit IMG outside Canada to work in underserved communities, the study results suggest that these licenses are also used to retain visa trainees [18, 19]. From a physician recruiter’s perspective, retaining a visa trainee who has trained in Canada may be an attractive alternative to recruiting an IMG with limited prior knowledge of Canada’s health care system or cultural components of care.

The proportion of visa trainees who worked in Canada in 2015 (53.6%) is more than double the proportion of visa trainees who worked in Canada in the 2 years following their training (24.0%). We are unable to determine whether visa trainees worked in their home country before returning to Canada or whether they were working in Canada but not included in Scott’s Medical Database. We found a similar pattern among non-visa IMG [17]. Given that non-visa IMG have no requirement to leave Canada, we suspect that many new-to-practice physicians, including visa trainees, may not opt to be listed in Scott’s Medical Database, especially if they take short-term or locum positions when they first start practice.

More than half of the visa trainees in the study remained in Canada in 2015, a retention rate that is greater than the rates reported in previous analyses. Using CAPER data, Thurber [8] reported that 18.7% of visa trainees who completed their training between 1990 and 1996 were working in Canada 5 years after their training. Both studies used Scott’s Medical Database to identify work locations; the difference in reported retention rates may stem from the construction of the study cohorts. While Thurber examined visa trainees who exited their PGME training, we followed a cohort of visa trainees who first entered either a family medicine PGME program between 2005 and 2009 or a specialty PGME program in 2005 or 2006. Also, retention rates may have changed over time. Thurber’s results related to visa trainees who exited training in the early 1990s while our study examines visa trainees who exited their programs between 2005 and 2012.

The World Health Organization’s Global Code of Practice of International Recruitment of Health Personnel calls upon member states to contribute to the development of health personnel in source countries while also respecting the right to mobility of health workers [20]. Our study highlights the difficulty in realizing both these conflicting goals; while the visa trainee program provides training opportunities to strengthen source country health workforce, they simultaneously facilitate the migration of IMG to Canada. Research conducted in other countries reflect similar difficulties in regard to costs, an influx of physicians in the training country, and difficulties maintaining a balance between the needs of both the training and source countries [21, 22]. For example, a study from the USA reported that a significant proportion of visa trainees ultimately entered into permanent practice in the country, which is contrary to the original purpose of the visa exchange program [23]. The United Kingdom changed regulations for Medical Training Initiative visa holders, requiring them to leave the United Kingdom at the end of their training and to spend a minimum of 12 months outside the country before applying for a work permit to return to the United Kingdom [22]. Further research is needed to describe and evaluate other models of providing assistance to source countries to develop and retain their medical workforce.

The study has limitations. Only current year data is available for Scott’s Medical Database, so while we could examine current (2015) work locations for the study cohort, we relied on existing Scott’s Medical Database records held by CAPER for work locations prior to 2015. Unfortunately, CAPER did not have data for 2010 and 2014. To limit the impact of missing data, we examined the first 2 years after completing PGME training to determine whether IMG were working in Canada. However, because of missing data, we could not use survival analyses to examine the length of time an IMG stayed in a work location.

The National IMG Database includes data from 2005 to 2011, a period of 7 years. Our sample size was therefore limited by the length of time required to complete PGME training and qualify for the MCCQE2 and specialty examinations. To ensure that we did not include IMG who may have written these examinations before 2005, we limited our sample to IMG who first entered PGME training during this period. While the inclusion criteria allow us to be confident about the qualifications of the IMG in the study, they limit the sample size thereby reducing statistical power. As noted above, a sizeable proportion of IMG exited their programs after 2011 and their examination may not have been included in the database. We have planned future work to examine visa trainees using alternate data sources, supplemented by primary data collection.

## Conclusions

Using administrative data held by CAPER, we found that 39.5% of visa trainees in the cohort passed the MCCQE2, 45.9% obtained a specialty designation, 24.0% worked in Canada following their training program, and 53.6% remained in Canada in 2015. While obtaining the credentials for a full license was a predictor of working in Canada, many visa trainees remained in Canada without them. These findings suggest that visa training programs represent another route for IMG to enter the physician workforce in Canada.

The growth in the number of visa trainees and the high retention of these physicians warrant further consideration of the oversight and coordination of visa trainee programs in provincial and in pan-Canadian physician workforce planning.

## Abbreviations

CAPER: Canadian Post-MD Education Registry
CFPC: College of Family Physicians of Canada
IMG: International medical graduate
MCC: Medical Council of Canada
MCCQE1: Medical Council of Canada Qualifying Examination Part 1
MCCQE2: Medical Council of Canada Qualifying Examination Part 2
PGME: Post-graduate medical education
RCPSC: Royal College of Physicians and Surgeons of Canada
